# Can consumption of raw vegetables decrease the count of sister chromatid exchange? Results from a cross-sectional study in Krakow, Poland

**DOI:** 10.1007/s00394-014-0697-9

**Published:** 2014-04-17

**Authors:** Aleksander Galas, Antonina Cebulska-Wasilewska

**Affiliations:** 1 Department of Epidemiology, Chair of Epidemiology and Preventive Medicine, Jagiellonian University Medical College, 7 Kopernika St, Kraków, Poland; 2 Department of Radiation and Environmental Biology, Institute of Nuclear Physics, Polish Academy of Sciences, 152 Radzikowskiego St, Kraków, Poland

**Keywords:** Biomarker, Sister chromatid exchange, Colorectal cancer, Controls, Raw and cooked vegetables, Fruits

## Abstract

**Background:**

Sister chromatid exchange (SCE) is a widely used sensitive cytogenetic biomarker of exposure to genotoxic and cancerogenic agents. Results of human monitoring studies and cytogenetic damage have revealed that biological effects of genotoxic exposures are influenced by confounding factors related to life-style. Vegetable and fruit consumption may play a role, but available results are not consistent. The purpose of the study was to investigate the effect of consumption of raw and cooked vegetables and fruits on SCE frequency.

**Methods:**

A total of 62 participants included colorectal cancer (CRC) patients, hospital-based controls and healthy laboratory workers. SCE frequency was assessed in blood lymphocytes. Frequency of vegetable and fruit consumption was gathered by structured semi-quantitative food frequency questionnaire.

**Results:**

SCE frequency was lowest among hospital-based controls (4.4 ± 1.1), a bit higher in CRC patients (4.5 ± 1.0) and highest among laboratory workers (7.4 ± 1.2) (*p* < 0.05). Multivariable linear regression showed a significant inverse effect (*b* = −0.20) of raw vegetable consumption, but not so for intake of cooked vegetables and fruits.

**Conclusions:**

The results of the study have shown the beneficial effect of consumption of raw vegetables on disrupted replication of DNA measured by SCE frequency, implying protection against genotoxic agents. Further effort is required to verify the role of cooked vegetables and fruits.

## Background

There is a general consensus that cancers develop as a consequence of an accumulation of DNA damage and a subsequent change in function of oncogenes and tumor suppressor genes. The DNA damage depends on type, concentration, and duration of exposure to carcinogens, and on effectiveness of cellular defense (e.g., antioxidants) as well as metabolic detoxification and repair mechanisms [1]. After exposure to genotoxic agents, DNA may present sister chromatid exchange (SCE).

SCE is a process whereby, during DNA replication, two sister chromatids break and rejoin with one another, physically exchanging regions of the parental strands in the duplicated chromosomes. This process is considered to be conservative and error-free, since no information is generally altered during reciprocal interchange by homologous recombination.

SCE is assessed and scored after the second S phase in the presence of the thymidine analog 5-bromodeoxyuridine (BrdU) [2]. The SCE assay is one of various methods in genetic toxicology and human population cytogenetic monitoring, being an indicator of disrupted replication or wrong chromatid segregation, and is frequently used as a sensitive cytogenetic biomarker of exposure to chemical genotoxic agents [3]. It is also considered as a sensitive measure of individual susceptibility to the effects of mutagens and also has been suggested as a possible indicator of an increased cancer risk [4].

SCEs appear as the consequence of so-called susceptibility, which is the effect of both the effectiveness of DNA repair mechanisms and the genotoxic effect of mutagens. The most common mechanism of genotoxicity is the generation of free radicals. Reactive oxygen species (ROS) may lead to formation of hydroxyl radicals, which, being highly destructive, result in direct damage to the DNA [5] in the form of strand breaks (single or double), oxidized purines and/or pyrimidines, as well as alkali labile sites [6], and these oxidative modifications have been observed in several conditions including cancer, cardiovascular disease and other age-related changes [7]. Several mechanisms have evolved that protect against effects of ROS. These include (1) enzymatic and non-enzymatic mechanisms that prevent formation of radicals, (2) pathways responsible for removal of radicals before damage occurs and (3) elimination of consequences of oxidative damage by either repair or elimination of damaged molecules, which all together prevent mutations [8].

It has been demonstrated that diet, especially plant components, has antioxidant properties. Diet rich in antioxidant nutrients may reduce the risk of several cancers [9]. However, in spite of extensive investigation of an effect of diet, especially of vegetables and fruits in the development of cancer, they are currently considered only as possibly or probably preventive [9]. Results are somewhat inconsistent, as a consequence of different study designs used and different end points considered [9–18]. Overall, a majority of case–control, some cohort and only a few intervention studies have supported a positive effect [9]. Reports from basic experimental research investigating the protective effect of dietary components on the DNA are also heterogeneous. There are studies showing beneficial effect of extracts from broccoli [10], dietary polyphenols [11] and a variety of dietary micronutrients [12]. Contrarily, there are also recent investigations showing no effect of consumption of vegetables and plant oil [13], brassica vegetables [14], vitamin C [15] and some other dietary components [16–18].

Nevertheless, fruits and vegetables are dietary components rich in vitamins and phytochemicals. Both are potentially protective and have shown effects by inhibition of DNA damage, cellular injury and degeneration [19, 20]. All in all, if fruits and vegetables are protective against DNA damage, this should be observed in vivo among different groups of individuals as a dose-dependent relationship between level of consumption and frequency of DNA damage or of endpoints related to DNA damage, such as SCEs.

## Purpose

The purpose of the study was to assess the association between consumption of vegetables and fruits and the frequency of SCEs across individuals diagnosed with colorectal cancer and cancer-free controls.

## Materials and methods

The cross-sectional investigation was carried out in 2011–2013 as a part of a larger case–control study. The design of the study has been described elsewhere [21, 22]. In brief, participants were individuals diagnosed with colorectal cancer (*n* = 22), some other acute chronic conditions (*n* = 16) and healthy laboratory workers (*n* = 24). Cases were patients newly diagnosed with sporadic (only) histologically confirmed adenocarcinomas of colorectal cancer treated at the I Chair of General Surgery and Department of Gastroenterological Surgery, Jagiellonian University Medical College, Krakow, Poland. In the second group, there were patients admitted to the University Hospital, Krakow, Poland, due to other cancer-unrelated conditions, and in the third-laboratory workers. After written consent had been obtained, participants were asked about basic characteristics and dietary habits including their average consumption of raw and cooked vegetables and fruits.

### Dietary questionnaire

Dietary habits were assessed by a semi-quantitative food frequency questionnaire (SFFQ) which had been developed in cooperation with the German Cancer Research Centre in Potsdam, where an introductory part of the European Prospective Investigation into Cancer and Nutrition (EPIC-Potsdam) project had been performed. In total, 148-dietary items were included in questions about consumption of cereals, dairy products, bread, type and cuts of meat and fish, fresh fruits (summer/winter time), salads and fresh and cooked vegetables, rice or pasta, soups, sweets, baked goods, drinks and others. For each food or beverage item, a commonly consumed portion size was specified by standardized photographs. Next, respondents were asked to provide information about frequency of consumption. For the research, information about usual (habitual) consumption over the period of 1 year by calendar seasons was gathered by trained interviewers. Patient cases were asked about their dietary patterns prior to the onset of gastrointestinal symptoms (if present) or prior to the beginning of the diagnosis process. The validity and reproducibility of the questionnaire was assessed and published [23]. Questions aimed to assess habitual consumption of vegetables and fruits have been provided in the “Appendix”. In the analysis, an average number of servings were analyzed. The size of a serving was standardized to the value of 80 g of eatable parts of fruits or vegetables.

For the subgroup of 62 individuals, blood samples were taken for an analysis of SCEs.

The study was conducted in accordance with the ethical principles of the Declaration of Helsinki and was approved by the Bioethical Committee of Jagiellonian University (number KBET/115/B/2011).

### Sister chromatid exchange assay

Blood samples were taken from all enrolled individuals and quickly transported to the laboratory unit. Lymphocytes were separated within an hour and cultured. All samples were incubated at 37 °C in RPMI 1640 medium with 20 % fetal calf serum, antibiotics and 0.075 mM BrdU. Then, lymphocytes were stimulated by phytohaemagglutinin (PHA) and cultured for 72 h. Two hours before the end of culturing, 0.1 µl/ml of colcemid solution (to stop dividing cells in metaphase) was added. Next cells were prepared following the standard procedure [24]. Slides were coded blindly. Finally, 50 well-spread second metaphases were analyzed for every participant.

*Proliferation rate index* was assessed from a distribution of cells scored in the first (*M*_1_), second (*M*_2_) and third (*M*_3_) division according to the following standard formula: PRI = (*M*_1_ + 2 × *M*_2_ + 3 × *M*_3_)/(*M*_1_ + *M*_2_ + *M*_3_). In order to determine PRI, a minimum of 100 consecutive metaphase cells per patient were evaluated. The number of cell cycles performed by each cell was determined, considering that when cells completed only one cell cycle (*M*_1_) both chromatids are labeled and all the chromosomes are uniformly strained. After second division (*M*_2_), DNA of one chromatid is labeled in every chromosome, showing a characteristic sister chromatid differentiation pattern. After three cell cycles (*M*_3_), approximately half of the chromosomes in a cell possess harlequin staining.

### Statistical analysis

Basic characteristics were presented as means and standard deviations, medians and interquartile ranges. Differences were tested by one-way ANOVA (analysis of variance) for normally distributed and by Kruskal–Wallis test for skewed variables. Categorized variables were tested by the chi-square test, or in a case of expected values of <5, by the Fisher’s exact test. For the comparison of SCEs across groups of vegetable and fruit consumption, the groups have been created by median-equal cutoffs. The fit to the normal distribution has been tested by the Shapiro–Wilk test, and, as the distributions fitted the normal distribution, the *t* test has been used. Linear regression was used to test the effect of consumption of vegetables and fruits on the SCE count. There were three main linear models investigated. First, a simple univariable model used to test the general pattern between dependent (SCE) and independent (vegetables or fruits) variables. Next, age and sex were used as covariates to verify the presence of relationship considering these two personal characteristic as main confounding variables; and finally, in the third model, we additionally used the diagnosis of colorectal cancer to account for cancer/non-cancer genetic susceptibility and vitamin supplementation (yes/no) as the SCE frequency might depend also on the antioxidative effect of some vitamins. Finally, all relevant variables were put together in one model. All analyses were performed using the statistical software package Stata/IC 11.2 for Windows, Stata Corp LP. A *p* value below 0.05 was considered statistically significant.

## Results

In total, 62 individuals were recruited and investigated in the study. There were three groups of individuals: 22 colorectal cancer patients, and in total 40 controls, including 16 hospital patients admitted due to acute conditions and 24 apparently healthy laboratory workers. The first two groups were part of a larger case–control study [21, 22] for which a subsample was randomly chosen for the SCE evaluation. Subsequently, a group of controls were enlarged by available blood samples of healthy laboratory staff.

Basic characteristics of the study participants are presented in Table 1. Groups varied significantly according to age (laboratory workers were younger), consumption of raw vegetables (highest amount among hospital-based controls, lowest in laboratory staff), vitamin supplementation (highest among laboratory staff, lowest among hospital controls).

**Table 1:** Basic characteristics of study participants

	CRC patients (*n* = 22)	Hospital-based controls (*n* = 16)	Laboratory staff (*n* = 24)	*p*
Age				
Mean (SD)	57.9 (10.1)	58.8 (12.6)	39.8 (12.7)	p* < 0.001
Median (Q1–Q3)	60.0 (49.0–66.0)	63.5 (52.5–67.5)	34.5 (28.0–52.5)	
Sex [*n*, (%)]				
Males	9 (40.9 %)	8 (50.0 %)	8 (33.3 %)	*df* = 2
Females	13 (59.1 %)	8 (50.0 %)	16 (66.7 %)	*p* ^chi^ = 0.573
Raw vegetable consumption (servings/week)				
Mean (SD)	6.6 (2.4)	6.9 (3.0)	5.0 (2.0)	p* = 0.023
Median (Q1–Q3)	7.0 (5.1–8.2)	6.1 (5.4–8.6)	5.0 (3.0–7.0)	
Cooked vegetable consumption (servings/week)				
Mean (SD)	3.7 (1.1)	3.3 (1.1)	4.0 (1.9)	*p* ^KW^ = 0.525
Median (Q1–Q3)	3.8 (2.7–4.6)	3.3 (2.5–4.0)	3.5 (2.0–5.0)	
Fruit consumption (servings/week)				
Mean (SD)	8.0 (4.1)	9.3 (6.5)	9.4 (5.6)	*p* ^A^ = 0.629
Median (Q1–Q3)	8.3 (4.5–9.9)	7.6 (5.5–11.9)	7.5 (5.0–15.0)	
Vitamin supplementation [*n*, (%)]	3 (13.6 %)	0	10 (41.7 %)	*p* ^F^ = 0.004
Smoker [*n*, (%)]				
No	15 (68.2 %)	10 (62.5 %)	19 (79.2 %)	*df* = 2
Yes	7 (31.8 %)	6 (37.5 %)	5 (20.8 %)	*p* ^chi^ = 0.491
SCE				
Mean (SD)	4.45 (0.99)	4.40 (1.11)	7.39 (1.23)	p** < 0.001
Median (Q1–Q3)	4.53 (3.48–5.13)	4.46 (3.72–5.16)	7.21 (6.59–8.32)	
PRI	*n* = 20	*n* = 10	*n* = 24	
Mean (SD)	2.38 (0.42)	2.36 (0.31)	1.95 (0.40)	p*** = 0.001
Median (Q1–Q3)	2.52 (1.99–2.67)	2.46 (2.29–2.54)	2.00 (1.62–2.25)	

*CRC* colorectal cancer, *HBC* hospital-based controls, *LS* laboratory staff, *SCE* sister chromatid exchange, *PRI* proliferation rate index, *df* degrees of freedom, *Chi* chi-square test, *KW* Kruskal–Wallis test, *A* one-way anova, *F* Fisher’s exact test

* One-way ANOVA, CRC versus LS *p* = 0.064; HBC versus LS *p* = 0.052

** One-way ANOVA, CRC versus LS *p* < 0.001; HBC versus LS *p* < 0.001

*** One-way ANOVA, CRC versus LS *p* = 0.002; HBC versus LS *p* = 0.026

Considering the average SCE frequency per cell, the highest was observed among laboratory workers (7.4 ± 1.2), next among CRC patients (4.5 ± 1.0) and the lowest in hospital-based controls (4.4 ± 1.1). Differences between laboratory workers and the two remaining groups were statistically significant. Otherwise, proliferation rate index (PRI) was significantly lowest in the group of laboratory staff (2.0 ± 0.4) as compared to that in the two other groups, and highest among CRC patients (2.4 ± 0.4) (Table 1).

As the main purpose of the investigation was to assess the role of vegetables and fruits, a linear regression model was use to assess the association between the aforementioned dietary components and SCE frequency. Vegetables were investigated as raw and cooked separately, and it was observed that consumption of raw vegetables was associated with a significant decrease in SCE frequency in either univariable (*b* = −0.21) or multivariable model (*b* = −0.14). Consumption of neither cooked vegetables nor fruits had a statistically significant effect on the SCE count (Table 2).

**Table 2:** Relationship between vegetable and fruit consumption and SCE frequency

Consumption (servings/week)	*b* ^1^	*p* ^1^	$R_{{{\text{model\,1}}}}^{2}$	*b* ^2^	*p* ^2^	$R_{{{\text{model\,2}}}}^{2}$	*b* ^3^	*p* ^3^	$R_{{{\text{model\,3}}}}^{2}$
Raw vegetables	−0.21	0.019	0.09	−0.22	0.003	0.45	−0.14	0.025	0.60
Cooked vegetables	0.21	0.192	0.03	0.20	0.119	0.38	0.20	0.060	0.59
Fruits	−0.003	0.941	0.000	0.001	0.976	0.36	0.01	0.694	0.56

Linear regression for the whole sample of *n* = 62

1—Univariable model: *b* ^1^—regression coefficient in univariable model, *p* ^1^ the *p* value for the univariable model, $R_{{{\text{model\,1}}}}^{2}$—the coefficient of determination of the univariable model

2—Multivariable linear regression, adjusted for age and sex: *b* ^2^—regression coefficient in the model, *p* ^2^—the *p* value for the model, $R_{{{\text{model\,2}}}}^{2}$—the coefficient of determination of the model

3—Multivariable linear regression, adjusted for the covariates from the model 2 and vitamin supplementation, and the diagnosis of CRC: *b* ^3^—regression coefficient in the model, *p* ^3^—the *p* value for the model, $R_{{{\text{model\,3}}}}^{2}$—the coefficient of determination of the model

Additionally, the effect of raw vegetable consumption on the SCE frequency was also observed in the fully adjusted model, i.e., adjusted for cooked vegetables, fruits, vitamin supplementation, age, sex and a diagnosis of CRC; as a result, the observed regression coefficient was *b*_SCE_ = −0.17 (*p* = 0.009; $R_{\text{model}}^{2}$ = 0.64; *p*_model_ < 0.0001) and, when the PRI count was added *b*_SCE_ = −0.20 (*p* = 0.016; $R_{\text{model}}^{2}$ = 0.71; *p*_model_ < 0.0001).

Finally, some comparisons across different levels of fruit and vegetable consumption (above and below median values) have been performed. These who consumed higher levels of raw vegetables presented lower levels of SCEs; however, differences were not statistically significant (Table 3).

**Table 3:** SCE count across groups of consumption (the cutoffs between low and high are medians)

	Level of raw vegetable consumption		
	Low <5.89 servings/week	High ≥5.89 servings/week	*p*
All groups together (*n* = 62)			
SCEs	(*n* = 31)	(*n* = 31)	
Mean (SD)	5.91 (1.77)	5.23 (1.84)	0.073
Median (Q1–Q3)	5.46 (4.28–7.45)	5.00 (3.50–6.60)	
CRC patients (*n* = 22)			
SCEs	(*n* = 9)	(*n* = 13)	
Mean (SD)	4.56 (0.88)	4.38 (1.08)	0.348
Median (Q1–Q3)	4.84 (3.91–5.23)	4.41 (3.48–4.97)	
Hospital-based controls (*n* = 16)			
SCEs	(*n* = 8)	(*n* = 8)	
Mean (SD)	4.60 (0.99)	4.21 (1.26)	0.252
Median (Q1–Q3)	4.24 (3.98–4.99)	4.83 (2.96–5.24)	
Laboratory staff (*n* = 24)			
SCEs	(*n* = 14)	(*n* = 10)	
Mean (SD)	7.53 (1.01)	7.18 (1.51)	0.246
Median (Q1–Q3)	7.51 (6.67–8.15)	6.79 (5.97–8.89)	

	Level of cooked vegetable consumption		
	Low <3.68 servings/week	High ≥3.68 servings/week	*p*
All groups together (*n* = 62)			
SCEs	(*n* = 31)	(*n* = 31)	
Mean (SD)	5.60 (1.59)	5.55 (2.05)	0.463
Median (Q1–Q3)	5.21 (4.28–6.93)	5.19 (3.91–6.85)	
CRC patients (*n* = 22)			
SCEs	(*n* = 10)	(*n* = 12)	
Mean (SD)	4.40 (0.79)	4.50 (1.16)	0.407
Median (Q1–Q3)	4.57 (3.50–4.97)	4.43 (3.44–5.21)	
Hospital-based controls (*n* = 16)			
SCEs	(*n* = 9)	(*n* = 7)	
Mean (SD)	4.70 (0.96)	4.03 (1.24)	0.121
Median (Q1–Q3)	4.28 (4.16–5.21)	4.65 (2.75–5.11)	
Laboratory staff (*n* = 24)			
SCEs	(*n* = 12)	(*n* = 12)	
Mean (SD)	7.27 (0.83)	7.50 (1.56)	0.332
Median (Q1–Q3)	7.21 (6.64–7.72)	7.47 (6.28–8.81)	

	Level of fruit consumption		
	Low <7.90 servings/week	High ≥7.90 servings/week	*p*
All groups together (*n* = 62)			
SCEs	(*n* = 31)	(*n* = 31)	
Mean (SD)	5.70 (2.05)	5.45 (1.58)	0.292
Median (Q1–Q3)	5.19 (4.28–7.45)	5.23 (4.20–6.67)	
CRC patients (*n* = 22)			
SCEs	(*n* = 10)	(*n* = 12)	
Mean (SD)	4.16 (0.83)	4.70 (1.07)	0.106
Median (Q1–Q3)	4.10 (3.48–4.84)	4.77 (3.91–5.18)	
Hospital-based controls (*n* = 16)			
SCEs	(*n* = 8)	(*n* = 8)	
Mean (SD)	4.53 (1.42)	4.28 (0.76)	0.330
Median (Q1–Q3)	4.95 (3.51–5.16)	4.18 (3.72–4.96)	
Laboratory staff (*n* = 24)			
SCEs	(*n* = 13)	(*n* = 11)	
Mean (SD)	7.61 (1.40)	7.12 (0.98)	0.169
Median (Q1–Q3)	8.10 (6.59–8.73)	6.96 (6.60–7.58)	

## Discussion

Our study showed a protective effect of raw vegetables measured by the frequency of SCEs. The effect was observed, when different individuals were considered as being a healthy person, or a non-cancer patient requiring hospitalization or a colorectal cancer patient. Across all these individuals, an increase in consumption of raw vegetables was associated with a decrease in the frequency of SCEs in blood lymphocytes. The negative relationship was rather stable, as it was observed even after adjustment for several covariates.

SCEs are used to measure individual effects of exposure to mutagens. Vegetables are a source of many biological compounds that are considered to be protective against DNA damage. Oxidative stress in a cell leads to the DNA oxidation, which is finally controlled by the repair of the DNA. The availability of antioxidants (e.g., antioxidative vitamins) can decrease the level of oxidative stress and finally decrease the frequency of DNA damage [25]. One of the vitamins with antioxidative properties is vitamin A (carotenoids) [26]. The effect of vitamin A was observed in animal studies, which showed an inhibition of SCE frequencies induced by some carcinogens [27, 28]. In vivo investigations have shown that retinoids decrease genotoxicity, metabolic activation and bindings to the DNA of many carcinogens such as aflatoxin B [29], *N*-nitrosamines [30] and dimethylbenz[a]anthracene [31]. There are also other compounds that may be responsible for the protective effect of vegetables such as vitamin C [32] or isothiocyanates [33] found in cruciferous vegetables. Although some studies failed to prove the protective effect of some dietary items such as vitamin C [8], this does not contradict our results. The purpose of our investigation was to assess the effect of vegetables as a whole, and we think that the protective effect of raw vegetables is related to the content of all beneficial dietary nutrients.

**Fig. 1:**
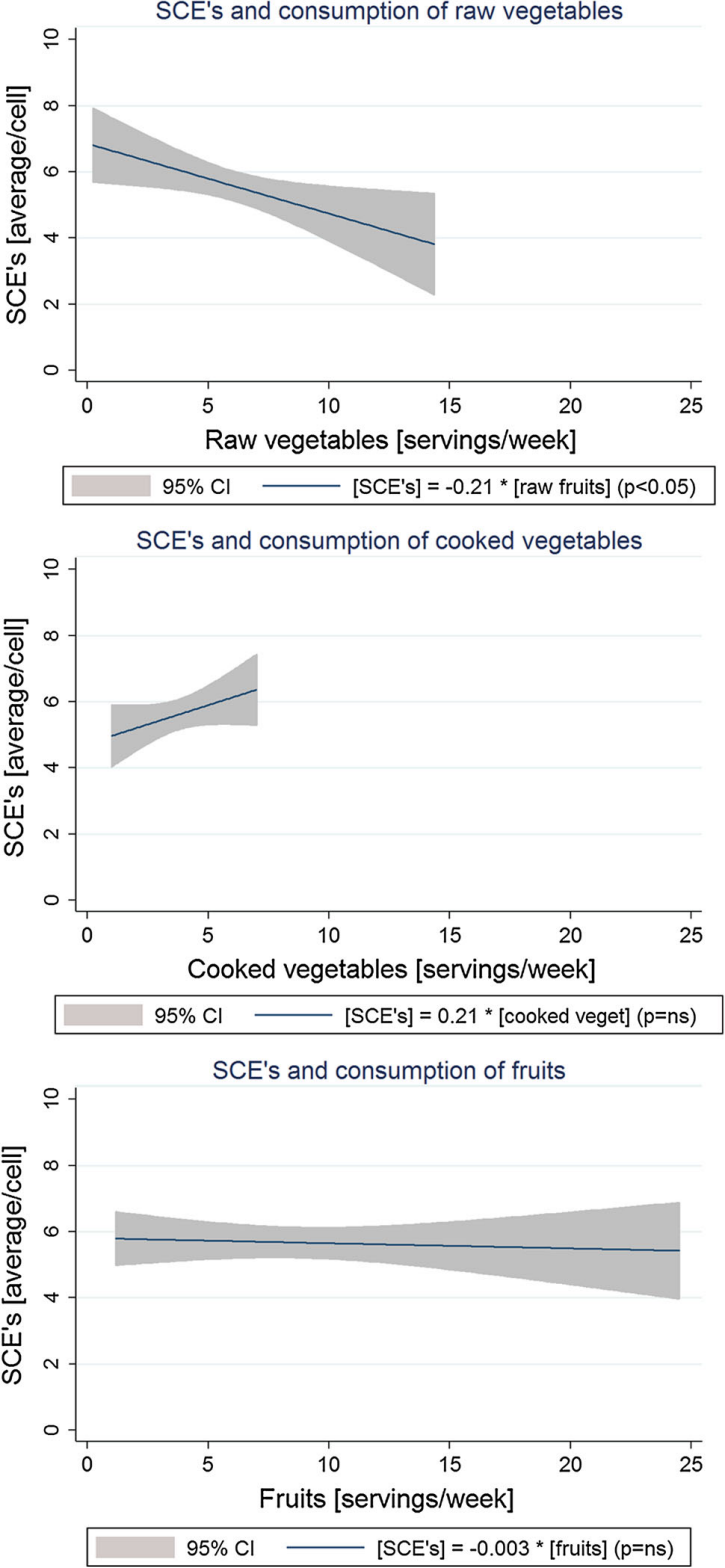
Consumption of vegetables and fruits and SCE frequency (linear regression with 95 % confidence intervals)

Our study failed to demonstrate any protective effect of cooked vegetables and fruits. There are some possible explanations. Firstly, in cooked vegetables, the content of vitamins and microelements is lowered after preparation [34]. Additionally, the range of consumption of cooked vegetables was very low in our study (Fig. 1). Finally, cooked vegetables may present a different dietary spectrum to raw vegetables, and thus, they may not be directly related to the decrease in SCE frequency. Regarding consumption of fruits, the effect was very weak, and statistically insignificant. At the moment, we cannot distinguish if this is an effect of a small sample size or, in fact, there is no relationship between number of fruit servings and the SCE frequency.

In our study, we did not observe a higher SCE frequency in CRC patients when compared to the two other that in control groups. In fact, the group of laboratory workers presented a higher SCE count. The finding with CRC patients is not surprising; as we analyzed blood lymphocytes for SCE frequency, and because we recruited for the study only sporadic CRC cases, their cancer risk was expected to be related mainly to the local tissue-related chromosomal instability and therefore was not observed at the level of the whole organisms. Similar results showing no differences between cancer cases and healthy controls were published before [35–37]. However, it is worth mentioning that study results are not consistent [38, 39] in this area.

In our study, the PRI was higher among colorectal cancer patients as compared to healthy laboratory workers, and also hospital-based controls presented higher PRI than the group of laboratory workers. The PHA-induced mitotic index in blood lymphocytes is a surrogate representing immune function and a potential of cell proliferation. Cell proliferation is associated with the pathogenesis of cancer [40] as it provides opportunities for genetic mutations. The proliferation of immune cells is a physiological process also observed in the presence of inflammation which was more likely to be observed among hospital-based controls. The differences in the PRIs between groups in our study provide information about proliferative potentials across groups and the lowest value observed in the “healthy laboratory workers” group suggests that they were indeed healthy at a time of investigation.

Regarding the group of laboratory workers, the participants in this group were of two different professions. There were analytical chemists (six people) described under discussion as “people who were continuously exposed to several chemicals”. As they were recruited voluntarily, there was a possibility that they perceived themselves as exposed to some risks and that is why they wanted to participate. The remaining 15 were also voluntarily recruited workers of the nuclear physics institute—apparently healthy with no signs or symptoms of a disease, but because of the nature of their work (some of them worked in laboratory units preparing slides of biological samples, and some others might be exposed to radiation as the exposure was present in some areas of the aforementioned institute)—they all together were named “laboratory workers”. Thus, there were very likely people who had been exposed to several risks, and therefore, they were considered as a separate group. Although it was not possible to measure chemical compound exposure at the workplace of these individuals, other studies reported that an increased count of SCE frequency was observed after occupational exposure to formaldehyde [41, 42], benzene [43] and polycyclic aromatic hydrocarbons [44], as well as among interventional cardiology laboratory workers [45] and among nurses handling cytostatic drugs [46].

### Possible limitations of the study

The presented study has also some limitations. One is its relatively small sample size across subgroups, and as a consequence any analysis performed in subgroups which were smaller than 40 individuals failed to show significant results.

Vegetables as well as fruits are very heterogeneous groups, with different amount of macro and micronutrients across items within the group. Thus, it is very difficult to answer which particular dietary components are responsible for the effect of raw vegetables. This is a point for discussion. We know that the size of the serving was standardized to achieve a kind of comparability in the content (“the amount of foods that provide a comparable amount of key nutrients from that food group”) between different types of fruits and vegetables [47, 48]; however, the use of a serving concept is a kind of a trade-off between the possibility to link a particular dietary component with a biological outcome and the necessity to create some guidelines regarding dietary habits recommended for a population. Thus, our study considered the possibility of the effect of a particular group of dietary items (recommended fruits and vegetables), but (due to relatively small sample size) we were not able to assess the effects of micronutrients (i.e., vitamins).

Secondly, there were studies showing higher frequency of SCE among smokers, as compared to non-smokers [49, 50], some with [50] and some without [49] correlation with a number of cigarettes smoked daily. In our study, smokers have a slightly higher SCE frequency as compared to non-smokers (means 5.7 vs. 5.5; medians: 5.2 vs. 5.1, *p* = 0.698), but this difference was not significant. There was also no difference in smoking frequency in CRC cases and hospital-based controls. Thus, we think that a distorting effect of smoking in the observed relationship between raw vegetables and SCE frequency in our study is rather unlikely. The high variability of vegetable consumption prevents us from investigating the role of vegetable subgroups. Moreover, diet may be also a source of heterocyclic amines. They are found in grilled fish and grilled meat, in juice from heated meat, and in stewed meat heated for a prolonged time [51]. As they are potentially mutagenic, the level of DNA damage depending on the level of exposure should be relatively low in our study, as only about 2 out of 38 of participants (for whom this information was available) consumed grilled meat on average more frequently than once per week.

The results of our study support the beneficial effect of consumption of a higher number of servings of raw vegetables; however, a problem may arise with regard to translating this information into practice. The SFFQ supported by standardized photographs of portion sizes was used to assess the size of a portion usually consumed. Results were recalculated into number of servings standardized to equal 80 g of eatable parts of vegetables (the same was for fruits). The calibration study, however, performed to assess the real level of consumption has shown that there is relatively high variability between the real size and the reported size of vegetables and fruits, and for the SFFQs prepared for the EPIC study (our questionnaire has been prepared with them), the real size of the serving of vegetables has been 72 g on average [52]. Nevertheless, even if the real size of a serving might be debatable, we believe that our study has shown the beneficial effect related to the number of servings of raw vegetables.

In summary, the results of our study performed across individuals with different characteristics have shown an inverse effect of consumption of raw vegetables on the damage of the DNA measured by SCE frequency. Thus, our study supports—on the cytogenetic level—epidemiologic investigations showing the beneficial role of raw vegetables. Further effort is required to determine the role of cooked vegetables and fruits.

## Appendix

The part of the semi-quantitative food frequency questionnaire used to gather information about habitual consumption of vegetables and fruits.

**Table:** .

Fresh fruits Summer/autumn	Not eaten	The portion size	My portion				Monthly		Weekly			Daily				Codes			
			1/2	1	2	3	1 time or less	2–3 times	1 time	2–3 times	4–6 times	1 time	2 times	3–4 times	5 or more times				
Coding	1		1	2	3	4	1	2	3	4	5	6	7	8	9				
Apple	□	One apple	□	□	□	□	□	□	□	□	□	□	□	□	□	□	□	□	035
Pear	□	One pear	□	□	□	□	□	□	□	□	□	□	□	□	□	□	□	□	036
Peach, nectarine	□	One piece	□	□	□	□	□	□	□	□	□	□	□	□	□	□	□	□	037
Wild cherries, cherries, mirabelle plums, plums	□	fig. 1	□	□	□	□	□	□	□	□	□	□	□	□	□	□	□	□	038
Grapes	□	fig. 2	□	□	□	□	□	□	□	□	□	□	□	□	□	□	□	□	039
Strawberries	□	fig. 3	□	□	□	□	□	□	□	□	□	□	□	□	□	□	□	□	040
Other berry fruits (currant, raspberry, blackberry, blueberry, others)	□	fig. 4	□	□	□	□	□	□	□	□	□	□	□	□	□	□	□	□	041
Bananas	□	One banana	□	□	□	□	□	□	□	□	□	□	□	□	□	□	□	□	042
Kiwi fruit	□	One piece	□	□	□	□	□	□	□	□	□	□	□	□	□	□	□	□	043

Fresh fruits Winter/spring	Not eaten	The portion size	My portion				Monthly		Weekly			Daily				Codes			
			1/2	1	2	3	1 time or less	2–3 times	1 time	2–3 times	4–6 times	1 time	2 times	3–4 times	5 or more times				
Coding	1		1	2	3	4	1	2	3	4	5	6	7	8	9				
Apple	□	One apple	□	□	□	□	□	□	□	□	□	□	□	□	□	□	□	□	044
Oranges, grapefruits	□	One piece	□	□	□	□	□	□	□	□	□	□	□	□	□	□	□	□	045
Bananas	□	One banana	□	□	□	□	□	□	□	□	□	□	□	□	□	□	□	□	047
Tangerine, kiwi fruit	□	One piece	□	□	□	□	□	□	□	□	□	□	□	□	□	□	□	□	046
Frozen strawberries (or other)	□	fig. 3	□	□	□	□	□	□	□	□	□	□	□	□	□	□	□	□	846

Salad/raw vegetables	Not eaten	The portion size	My portion				Monthly		Weekly			Daily				Codes			
			1/2	1	2	3	1 time or less	2–3 times	1 time	2–3 times	4–6 times	1 time	2 times	3–4 times	5 or more times				
Coding	1		1	2	3	4	1	2	3	4	5	6	7	8	9				
Heads of lettuce	□	fig. 1	□	□	□	□	□	□	□	□	□	□	□	□	□	□	□	□	069
Mixed salad (cucumber, tomato, pepper, etc.)	□	fig. 2	□	□	□	□	□	□	□	□	□	□	□	□	□	□	□	□	070
Cucumber salad	□	fig. 3	□	□	□	□	□	□	□	□	□	□	□	□	□	□	□	□	071
Carrot salad	□	fig. 4	□	□	□	□	□	□	□	□	□	□	□	□	□	□	□	□	073
Cabbage salad (excluding sauerkraut)	□	fig. 1	□	□	□	□	□	□	□	□	□	□	□	□	□	□	□	□	873
Radish or radish salad	□	fig. 3	□	□	□	□	□	□	□	□	□	□	□	□	□	□	□	□	072
Pepper	□	One piece	□	□	□	□	□	□	□	□	□	□	□	□	□	□	□	□	075
Raw tomatoes in summertime	□	One piece	□	□	□	□	□	□	□	□	□	□	□	□	□	□	□	□	076
Row tomatoes in wintertime	□	One piece	□	□	□	□	□	□	□	□	□	□	□	□	□	□	□	□	077
Chives, raw onion	□	One piece or a bunch	□	□	□	□	□	□	□	□	□	□	□	□	□	□	□	□	877

Please, remember to report only dishes made from raw vegetables

**Table:** .

Cooked vegetables	NOT eaten	The portion size	My portion				Monthly		Weekly			Daily				Codes			
			1/2	1	2	3	1 time or less	2–3 times	1 time	2–3 times	4–6 times	1 time	2 times	3–4 times	5 or more times				
Coding	1		1	2	3	4	1	2	3	4	5	6	7	8	9				
Cooked cabbage (white or red)	□	fig. 1	□	□	□	□	□	□	□	□	□	□	□	□	□	□	□	□	078
Cauliflower	□	fig. 1	□	□	□	□	□	□	□	□	□	□	□	□	□	□	□	□	878
Brussels sprout	□	fig. 1	□	□	□	□	□	□	□	□	□	□	□	□	□	□	□	□	879
Cooked spinach	□	fig. 2	□	□	□	□	□	□	□	□	□	□	□	□	□	□	□	□	082
Cooked carrot	□	fig. 1	□	□	□	□	□	□	□	□	□	□	□	□	□	□	□	□	084
Cooked beetroot and other root vegetables, e.g., cooked celeriac	□	fig. 1	□	□	□	□	□	□	□	□	□	□	□	□	□	□	□	□	085
Salad made of cooked vegetables	□	fig. 1	□	□	□	□	□	□	□	□	□	□	□	□	□	□	□	□	089
Beans (including yellow beans, broad beans), green beans, beans with carrot	□	fig. 2	□	□	□	□	□	□	□	□	□	□	□	□	□	□	□	□	088

Even if some of vegetables you may consume in some seasons of the year, please, report the average for the whole calendar year
